# Changes in P-glycoprotein activity are mediated by the growth of a tumour cell line as multicellular spheroids

**DOI:** 10.1186/1475-2867-5-20

**Published:** 2005-07-07

**Authors:** Ponce de León Valeria, Barrera-Rodríguez Raúl

**Affiliations:** 1 Depto. de Bioquímica. Instituto Nacional de Enfermedades Respiratorias-SSA México. Clza. Tlalpan, 4502, C.P. 14080, México, D.F

**Keywords:** P-Glycoprotein, Multicellular spheroids, Multicellular drug resistance, NSCLC

## Abstract

**Background:**

Expression of P-glycoprotein (P-gp), the multidrug resistance (MDR) 1 gene product, can lead to multidrug resistance in tumours. However, the physiological role of P-gp in tumours growing as multicellular spheroids is not well understood. Recent evidence suggests that P-gp activity may be modulated by cellular components such as membrane proteins, membrane-anchoring proteins or membrane-lipid composition. Since, multicellular spheroids studies have evidenced alterations in numerous cellular components, including those related to the plasma membrane function, result plausible that some of these changes might modulate P-gp function and be responsible for the acquisition of multicellular drug resistance. In the present study, we asked if a human lung cancer cell line (INER-51) grown as multicellular spheroids can modify the P-gp activity to decrease the levels of doxorubicin (DXR) retained and increase their drug resistance.

**Results:**

Our results showed that INER-51 spheroids retain 3-folds lower doxorubicin than the same cells as monolayers however; differences in retention were not observed when the P-gp substrate Rho-123 was used. Interestingly, neither the use of the P-gp-modulating agent cyclosporin-A (Cs-A) nor a decrease in ATP-pools were able to increase DXR retention in the multicellular spheroids. Only the lack of P-gp expression throughout the pharmacological selection of a P-gp negative (P-gp^neg^) mutant clone (PSC-1) derived from INER-51 cells, allow increase of DXR retention in spheroids.

**Conclusion:**

Thus, multicellular arrangement appears to alter the P-gp activity to maintain lower levels of DXR. However, the non expression of P-gp by cells forming multicellular spheroids has only a minor impact in the resistance to chemotherapeutic agents.

## Background

Multidrug resistance to chemotherapy is one of the biggest problems in the treatment of cancer. Currently, the best understood mechanism of multidrug resistance (MDR) is associated with the overexpression of protein efflux-pump known as P-glycoprotein (P-gp), but other non-Ppg mechanisms are also involved (i.e. MRP1, topoisomerases, glutathione-S transferases, etc). The P-gp is the protein product of the MDR-1 gene and is expressed as a transmembranal protein (Mr 170 000) capable of decreasing the intracellular concentration of a broad range of cytotoxic agents in an energy-dependant mediated efflux [1,2]. Overexpression of P-gp in human cell lines confers resistance to many of the most effective chemotherapeutic agents used clinically in chemotherapy, including anthracyclines (e.g., doxorubicin (DXR)), *Vinca *alkaloids (e.g., vincristine), epipodophyllotoxins (e.g., etoposide), actinomycin D, paclitaxel, as well as many others non-chemotherapeutic agents like Rhodamine-123 and ethidium bromide [3]. Although the more accepted model is that the P-gp by itself extrude chemotherapeutic agents out of the cells, more recent studies suggest that P-gp activity may be modulated by cellular components such as membrane proteins, membrane-anchoring proteins or the composition of lipids themselves [4-7].

Since the first studies by Sutherland et al in 1979 [8], it was shown that tumour cells growing as multicellular spheroids resembles many of the behaviours found in solid tumours, including multicellular resistance (MCR) [9,10]. Using the model of multicellular spheroids, several authors have shown that P-gp, is more efficient to conferring resistance in cells cultivated as spheroids as compared to cells cultivated as monolayers [11-13]. From these observations, one question arises: Can tumour cells modulate its P-gp activity as a direct consequence of the environmental condition where grown? To address this question, we studied an NSCLC cell line named INER-51 that showed a P-gp-mediated resistance to DXR in the spheroid model. In early studies with INER-51 cells, we found that the formation of multicellular spheroids does not show any increase in mRNA for MDR-1 gene or in a differential P-gp expression in the specific areas of the spherule.

## Results

### Doxorubicin and Rhodamine-123 retention in spheroids

**Figure 1:**
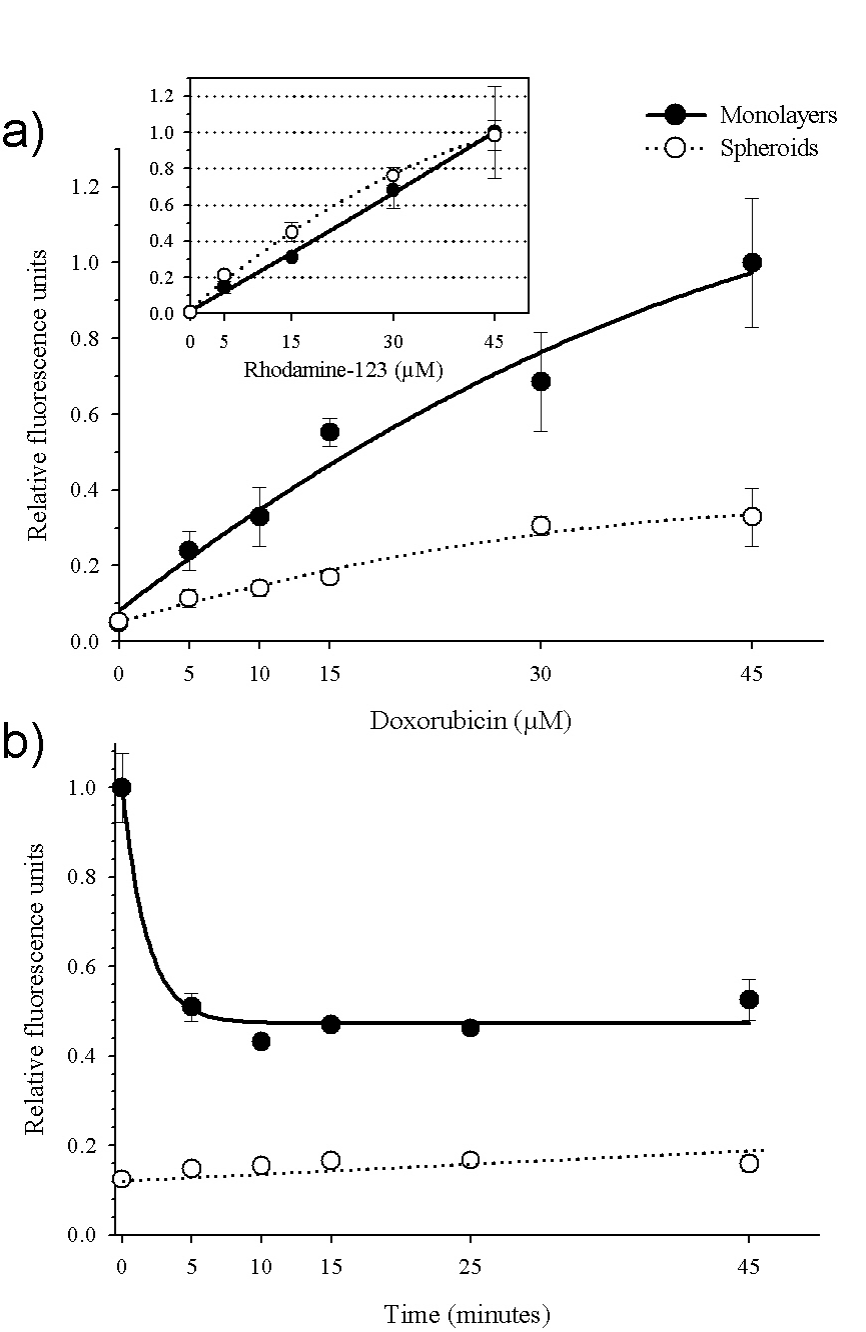
Dose-dependence of doxorubicin-retention in INER-51 cells. (a) Tumour cells growth as monolayers (*filled circles*) or multicellular spheroids (*open circles*) were incubated with increased concentrations of DXR or Rho-123 (*Insert box*) for 30 min and then intracellular fluorescence was determinated by spectrofluormetry. b) Time-course of DXR efflux in INER-51 cells. Monolayers (*filled circles*) and a multicellular spheroids (open circles) were previously loaded with 30 μM of DXR for 30 min and afterwards the remaining DXR was quantitated in several intervals of time. Each point represents the mean of at least 3 experiments and error bars are the standard error of the mean.

It is well known that multicellular spheroids are more resistant to chemotherapeutic drugs than the same cell cultures as monolayers [11,12]. With the aim to evaluate differences in P-gp activity in multicellular spheroids with respect to monolayers, two P-gp substrates were used (DXR and Rho-123). Both compounds were chosen, because they are good P-gp substrates with an autofluorescence capacity. Our results showed that in monolayers the amount of DXR retained in the cells was in direct proportion to the drug added to the medium (Figure 1). In contrast, multicellular spheroids showed a lower capacity for DXR retention (3-fold lower) than monolayers. Interestingly, this poor retention was not observed when Rho-123 was used as P-gp substrate because the Rho-123 levels retained were equal in both types of cultures (**box insert in **Figure 1a).

Since the drug-retention is a dynamic process that involves drug uptake (simple diffusion) as well as drug linkage (diffusion vs. expulsion mediated for an active mechanism), we tried to determine whether a more efficient P-gp-dependent drug removal was responsible for allowing lower intracellular DXR concentrations. Thus, drug-removal in monolayers previously loaded with 30 μM of DXR during 30 min showed a first order decay reaction with a half-time of drug concentration at the first 5 minutes interval that indicated the presence of an active transport mechanism (Figure 1b). However, for multicellular spheroids the presence of active transport was not evident since its was not possible to load the cells with sufficient amounts of DXR to determine the efflux values. A common observation in these experiments was that a constant amount of DXR was retained for a longer period of time (45 minutes) in cells grown as monolayers. A possible explanation could be from the presence of positively charged-DXR, which stores in acidic vesicles as chromaffin granules and lysosomes [20,21].

### Circumvention of DXR retention with P-pg reversal agents

**Figure 2:**
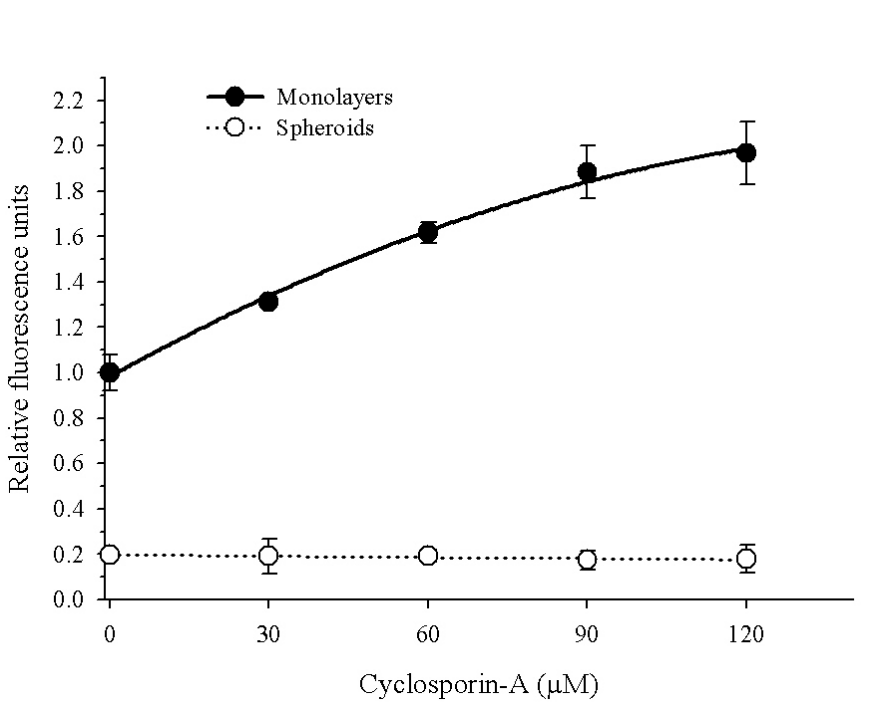
Circumvention of doxorubicin-retention by cyclosporin-A (Cs-A). INER-51 cells growth as monolayers (*filled circles*) or multicellular spheroids (*open circles*) were treated with increase concentrations of Cs-A for 1 hour previous to being loaded with 30 μM of DXR for 30 min in presence of reversal agent. Doxorubicin retained was determinate by spectrofluormetry. Each point represents the mean of at least 3 experiments and the error bars are the standard error of the mean.

With the aim to determine how much the P-gp influences the levels of DXR retained, the modulator agent Cs-A was employed. As shown in the Figure 2, the incubation of monolayers with 5 μM of Cs-A efficiently enhances (2-fold) the intracellular DXR fluorescence in direct relation to reversal agent concentration. Surprisingly, the P-gp-modulation activity of Cs-A was not evident in multicellular spheroids because no effect in the intracellular DXR retention could be seen. Also, other reversal agents, like SDZ PSC 833 and verapamil were not able to increase DXR retention, neither in monolayers nor in multicellular spheroids (*data not shown*).

### Effect of ATP-depletion in DXR retention

**Figure 3:**
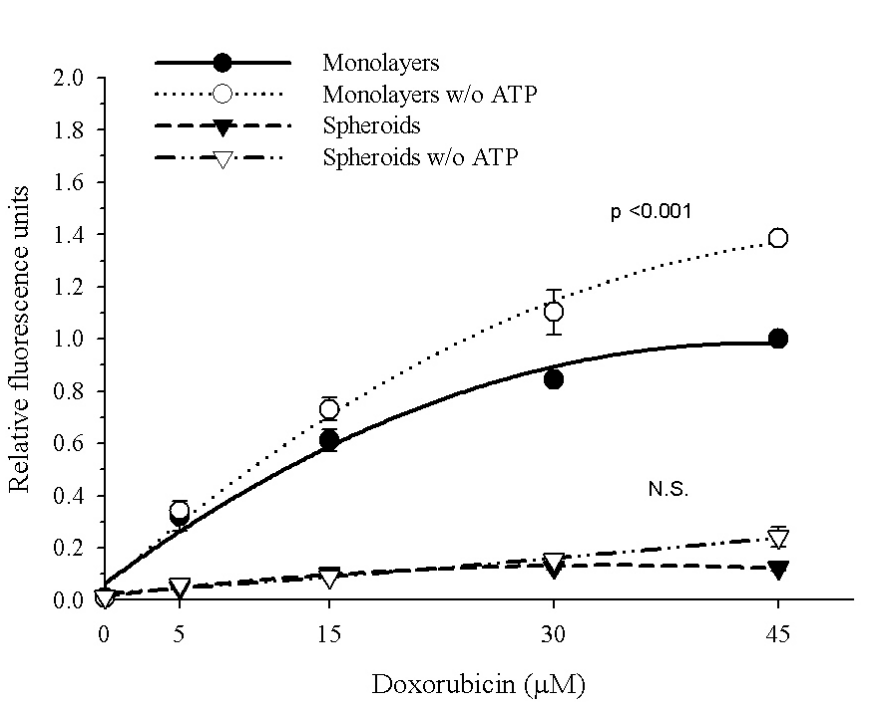
Effect of ATP-depletion in doxorubicin-retention. INER-51 cells as monolayers or multicellular spheroids were pre-incubated (*open symbols*) or not (*filled symbols*) with 1 mM sodium-cyanide, 10 mM sodium-fluoride, 10 mM sodium azide before the addition of increased DXR concentrations. The intracellular DXR-fluorescence was evaluated by spectrofluormetry. Each point represents the mean of at least 3 experiments and the error bars are the standard error of the mean. The Student's *t*-test for paired data was performed to identify changes in DXR retention. The results were considered to be significant when p < 0.05. *NS*: no significant.

P-gp is a protein that belongs to the ABC binding cassette protein, for which efficient drug efflux needs ATP hydrolysis. Thus, with the propose to achieve more information about the P-gp function, we decided to inhibit the P-gp activity through depletion of ATP-pools. Three metabolic poisons were used to be sure of complete ATP-depletion in multicellular spheroids. Figure 3 shows that ATP-depletion did induce a 1.5-fold increase of DXR retention in cells maintained as monolayers but none effect was again seen when in ATP-depleted multicellular spheroids.

### Achieving the PSC-1 mutant clone non-expressing P-gp

**Figure 4:**
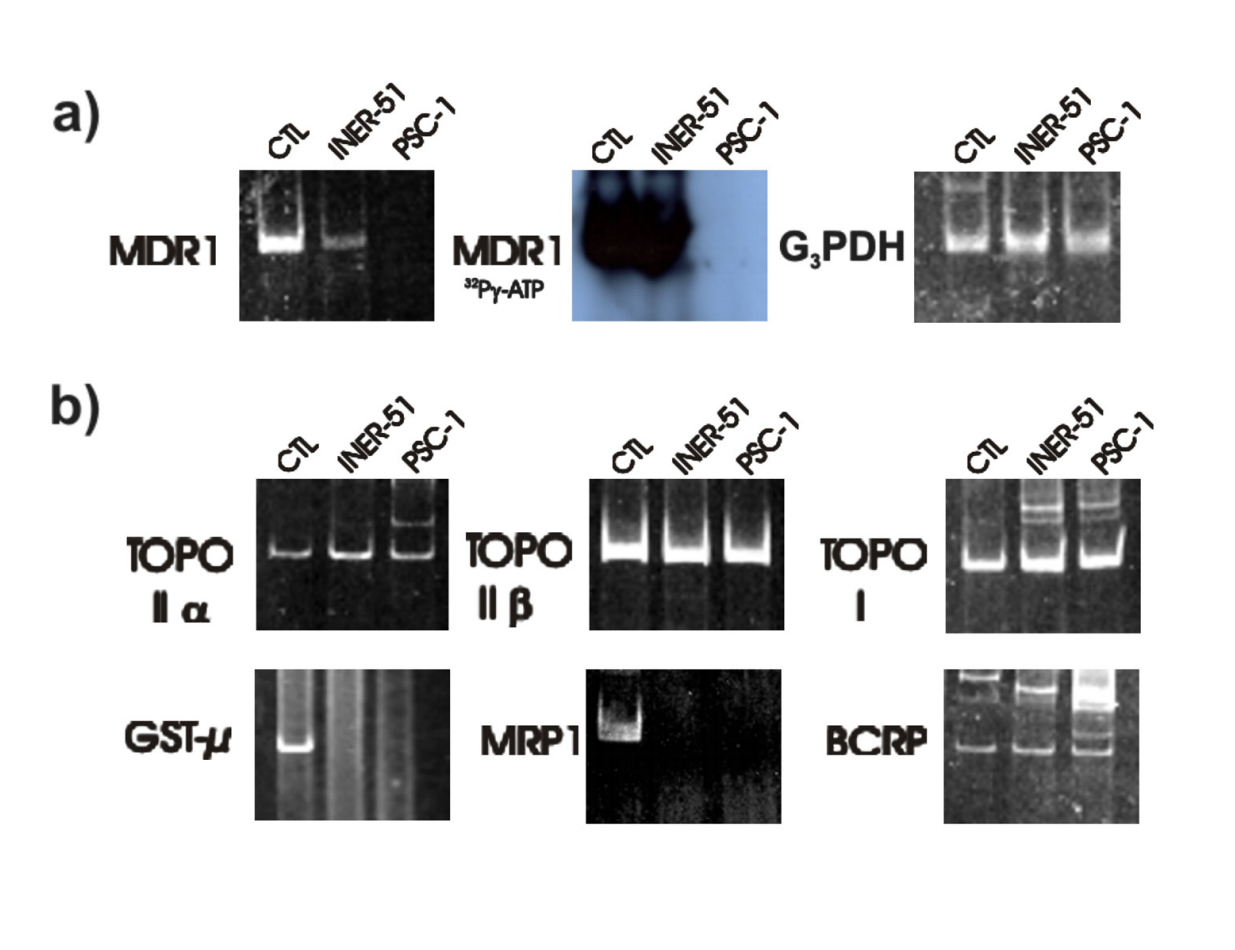
RT-PCR analysis of the levels of expression for MDR genes in the parental cell line INER-51 as well as the PSC-1 cell clone. a) Amplification of MDR-1 gene visualized by ethidium bromide (left) or autoradiagraph (middle). The expression of G_3_PDH gene was used as a constitutive control for the integrity of the RNA molecules. b) Amplification by RT-PCR of other non-MDR-1 genes related to MDR phenotype. As control (CTL) of gene expression, the next cell lines and tissues were used: INER-37 cell line for MRP1 and GST-μ; A427 cell line for MDR-1; HeLa cell line for topoisomerase I, topoisomerase IIα and topoisomerase IIβ; finally, placental tissue was used as a control of BCRP expression.

With no possibility of obtaining irrefutable evidence of P-gp modulation from multicellular spheroids, we decided to eliminate the P-gp expression from the lung cancer line. Therefore, through the co-selection of the parental cell line INER-51 with 5 μM of DXR and 5 μM of SDZ PSC 833, we were able to obtain one mutant cell clone that did not express P-gp, which was named PSC-1 line (Figure 4a). *In vitro *RT-PCR analysis of other MDR-related genes did not show qualitative differences between parent and mutant cells (Figure 4b). The amplification experiments showed that both cells lines express with approximately with the same intensity transcripts for topoisomerase I, topoisomerase IIα and topoisomerase IIβ but neither of them expressed the multidrug resistance-associated protein (MRP1) or glutathione-S transferase-μ.

### DXR retention and drug cytotoxicity in PSC-1 cells

**Figure 5:**
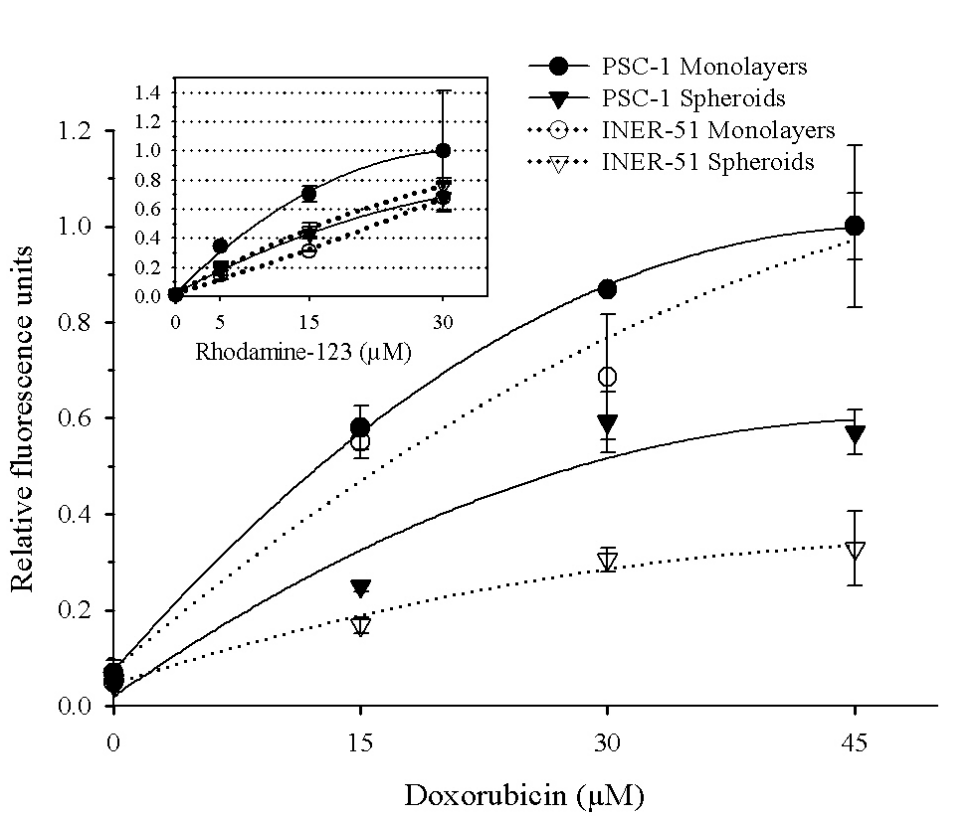
Comparative doxorubicin retention of PSC-1 cells and INER-51 cells. PSC-1 tumour cells growth as monolayers (*filled circles*) or multicellular spheroids (*filled triangles*) and INER-51 monolayers (*open circles*) or multicellular spheroids (*open triangles*) were incubated with increased concentrations of DXR (as in Figure 1) and then intracellular fluorescence was determined by spectrofluormetry. *Insert box*. Rhodamine-123-retention in PSC-1 cell growth as monolayers or multicellular spheroids.

The similarities between INER-51 cells (P-gp^pos^) and PSC-1 cells (P-gp^neg^), allowed us the possibility to evaluate whether or not the lower DXR-retention levels were mediated by a positively modulated P-gp-mechanism. The incubation of PSC-1 spheroids with increasing DXR concentrations showed a significative increase in the drug retention (1.8-fold) in comparison to INER-51 spheroids and was only 1.6-fold lower than PSC-1 growth as monolayers (Figure 5). When Rho-123 was assayed, an increase in the retention of the dye relative to the parental line INER-51 as monolayers was evident. In PSC-1 spheroids, the levels of Rho-123 retention were similar in both INER-51 monolayers and multicellular spheroids (**box insert in **Figure 5).

**Figure 6:**
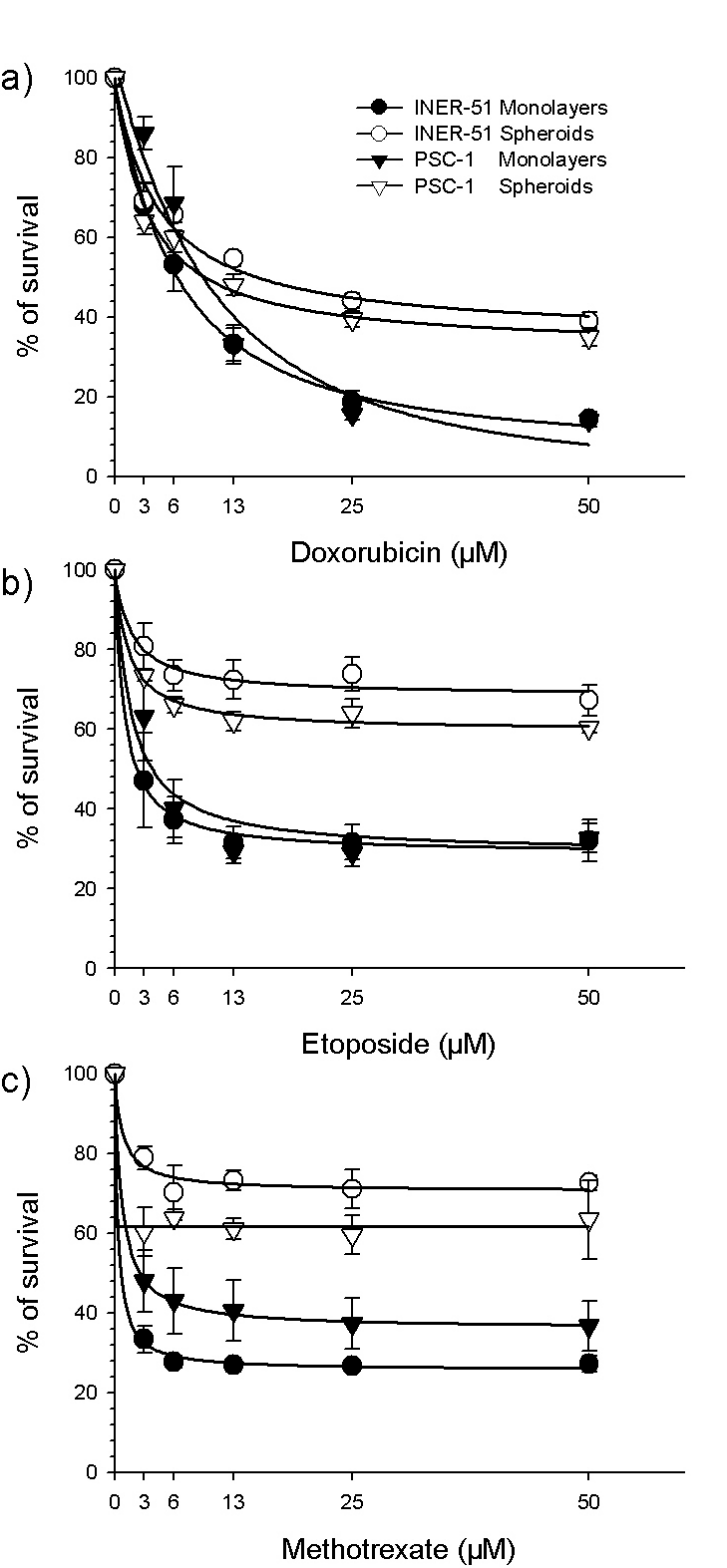
Cytotoxicity of doxorubicin (a), etoposide (b) and methotrexate (c) in monolayers or multicellular spheroids from PSC-1 (P-gp^neg^) clone cells in comparison to the parental INER-51 cell line. Cytotoxicity was measurement by the MTT colorimetric assay and expressed as percent growth inhibition in comparison with the untreated tumour cells. Each point represents the mean of at least 3 independent experiments and error bars are the standard error of the mean

Several techniques have been used to evaluate the MCR in multicellular spheroids. Since in previous experiments we were unable to successfully disaggregate multicellular spheroids through trypsin-based protocols, we decided to evaluate the MCR using the MTT assay (which as been previously evaluated by Furukawa et al[17]. Interestingly, this assay showed that the increase in DXR retention had only a minor impact in the MCR. Thus, PSC-1 spheroids were able to maintain their resistance to DXR or etoposide as the parental INER-51 cells (Figure 6a,b) with only minor changes in the IC_50 _values (Table 1). Only when PCS-1 cells were assayed against the cytotoxic effect of methotrexate (which use another detoxification via previous to P-gp), differences between INER-51 cells and PSC-1 cells could be seen (Figure 6c).

**Table 1:** Indices of cytotoxicity (IC_50_) to several cancer drugs founded in multicellular spheroids and monolayer.

Drug	Monolayers IC_50 _(μM)			Multicell. spheroids IC_50 _(μM)		
	**INER-51**	**PCS-1**	*RR*	**INER-51**	**PCS-1**	*RR*
Doxorubicin	6.2	9.2	1.4	15	9.8	0.6
Etoposide	2.2	4.1	1.8	>50	>50	----
Methotrexate	0.8	2.8	3.5	>50	>50	----

Drug exposition was continuous for 72 hours and drug cytotoxicity was performed by MTT assay. IC_50_: Fifty percent of the decrease in the survival rate. RR: The value of relative resistance of INER-51 cells vs PSC-1 cells as monolayers or multicellular spheroids.

## Discussion

It is well known that the three-dimensional arrangement evoke deeper structural changes in the cells to maintain the integrity of the multicellular structure, some of which include: a) the expression of proteins of ECM [22]; b) membrane protein anchoring [23,24]; c) heat shock proteins [25] and d) adhesion proteins as well as changes in lipid membrane composition and hypoxia [26-29]. Another phenomenon frequently observed in tumour cells growing as multicellular spheroids is the acquisition of MCR. In spheroids cultures as well in monolayer systems one of the major mechanisms to confer resistance is shown by the expression of P-gp. However, some evidence suggests that to confer MCR, the P-gp also seems to work more efficiently [11,12]. Recent data suggests that P-gp activity can be modulated through the interaction with diverse cellular components, some of which are also altered when cells are multicellular spheroids [4-7,11,12]. Thus, it would be interesting to know if to acquire MCR, tumour cells spheroids can modulate its P-gp activity.

Our findings suggest that a more efficient P-gp-mediated efflux seems to be responsible for maintaining lower levels of DXR in INER-51 spheroids than cells in monolayer cultures. However, this P-gp mediated drug efflux did not seem to operate under the same conditions with others substrates, because when the lipophilic cation Rho-123 was used, the levels of retention were similar and independent of the culture conditions.

Under the spheroid condition, P-gp appears to obey different regulatory mechanisms since neither Cs-A treatment nor ATP-depletion were able to increase the levels of DXR into the spherules. Other non-P-gp mediated mechanisms also appear to be operating to maintain lower DXR levels in the multicellular spheroids, because the lack of P-gp expression did not reach levels of DXR comparable to the monolayer's cultures and had only a minor impact in the acquisition of MCR to chemotherapeutic agents, including DXR.

Some evidences has shown that the membrane-cell composition can modulate the transbilayer movement rate of MDR-type drugs across the membrane and consequently affects the "competition" between the active P-gp-mediated drug efflux and the passive drug uptake: i.e. retention [30]. Particularly, DXR has shown to have a lower rate of penetration through membranes due to its specific interactions with cardiolipin [31,32]. Since membrane changes affecting plasma membrane do not confer resistance themselves but could drive the P-gp function [33-35], we hypothesize a possible drawing to understand how P-gp successfully maintains lower DXR concentrations. In this picture, a delay in the rate of passive transbilayer movement of DXR through the plasma membrane results in an enhanced ability of P-gp to recognize and extrude it out of the spheroids. Thus, when P-gp is absent (as in PSC-1 cells), the saturation of lipid targets permit the increase of the DXR concentration into the multicellular spheroids. Enhanced efficiency of P-gp to extrude substrates has been fully demonstrated by different members of the DXR and rhodamine dry analogues, with each one having different lipophilic properties [36].

Several studies have shown that the use of modulator agents provoke the sensitisation of cell growth as multicellular spheroids [37-39]. The efficacy of these agents is due to the higher permeability rate in relation to P-gp substrates [40]. However, when INER-51 spheroids were pre-treated with 10 μM of the modulator agent of Cs-A, no increase in DXR retention was observed. A similar phenomenon was observed in multicellular spheroids treated with verapamil [38,39]. Today, a number of studies using various techniques have suggested a probable model of interaction between P-gp-substrates with modulators agents and how these molecules bind to different sites on P-gp [41]. Four binding sites have been identified on P-gp of which vinblastine and daunorubicin bind to the site I, whereas modulators bind to site IV. In this model, all the binding sites display allosteric interaction between each one of them, thus affecting P-gp-mediated activity. In addition, an unrecognised binding site has been proposed for Rho-123 [42-44]. Thus, the failure of Cs-A to inhibit P-gp-mediated DXR efflux may be a consequence of changes in the P-gp affecting the modulator-binding site or the allosteric inhibition with some hypothetic molecule. Recently, in cells with MDR-phenotype, it has been demonstrated that a specific interaction exists between P-gp and HSP-90 [45]. If spherules formation elicits the expression of HSP-90, which is able to bind P-gp, the failure of Cs-A inhibition might be understood. Furthermore, the allosteric inhibition of the modulator site by unknown factors could be an explanation for the common failure seen when solid tumours, including lung cancer tumours, are treated with reversal agents [46].

Another interesting observation came from the impact of ATP depletion in DXR retention. For this experiment we used a metabolic cocktail poison to be certain of the ATP pools depletion. However, this treatment was unable to increase DXR retention in multicellular spheroids but its effect was evident in monolayers. Since, there is a general consensus in the literature that P-gp is an energy-driven pump, where the energy is provided by the hydrolysis of ATP, our data are puzzle. Nevertheless, using competitions assays, Biswas EE [47], demonstrated that NBD1 of histidine permease (*Hisp*) and maltose transporter protein (*Malk*), two ABC members, can function as a general nucleotide binding domain, with a nucleotide preference CTP>GTP>ATP>>UTP. Thus, the impact of ATP depletion in DXR retention is not understood for the moment.

In recent years, it has become clear that the multiple non-P-gp mediated mechanisms may be operational in tumour spheroids to confer MCR [48]. One mechanism most recently described is the breast cancer resistance protein (BCRP), which is a drug pump efflux able to efficiently extrude DXR [49]. Since INER-51 cells express mRNA for BRCP, we cannot discard the activity of this protein as a mechanism that helps to maintain lower DXR retention levels. However, the activity of BCRP as pump efflux is higher for Rho-123 efflux than DXR, although their contribution to the drug retention in spheroids is controversial [50].

In addition to DXR resistance, INER-51 spheroids also showed resistance to other anticancer drugs such as etoposide and methotrexate. Commonly, the etoposide-resistance involves alterations in the nuclear target enzymes topoisomerases. Oloumi et al[51] indicate that alterations in subcellular localization of topoisomerases type II may have an important role in resistance to cytotoxic agents when cells are in a close contact. Whereas Luo et al[52] showed that phosphorylation of topo II alpha was reduced at least 10-fold in the outer cells of V79 spheroids relative to monolayers. However, the role of topoisomerase II alpha as a mechanism to confer MCR to etoposide in INER-51 spheroids has not been evaluated yet and will be considered for future experiments.

A different situation can be speculated for the resistance to methotrexate. Under physiological conditions, the weak acid methotrexate tends to be in the charged form and is taken up into cells largely by a folate transport mechanism. Unlike DXR, methotrexate is not sequestered in membranes nor in acidic endosomes, but it may be "trapped" inside cells by polyglutamation [53]. Thus, the differences in methotrexate resistance of INER-51 cells relative to PSC-1 cells could arise from differences in P-gp-independent metabolic pathways.

Finally, several reports have shown a lower MDR-1 expression in NSCLC [54]. However, some authors have shown an important role of P-gp expression in lung cancer tumours and particularly in lung cancer diagnosed in people whom smoke tobacco [55-57]. Due to our findings, it would be interesting to evaluate both the levels of P-gp expression as well as P-gp activity in lung cancer tumours.

## Conclusion

Our results suggest that in INER-51 cells cultured as multicellular spheroids, a more efficient P-gp activity is responsible for maintaining lower retention levels of DXR in comparison to the P-gp activity of cells grown as monolayers. Interestingly, whereas the P-gp expression helps maintain lower levels of DXR in the multicellular spheroids, the mechanisms that govern the MCR seems to be different because the lack of P-gp-expression only showed a minor impact of resistance to several chemotherapeutic drugs, suggesting that other non-P-gp mechanisms are also operating.

## Methods

### Chemicals

Doxorubicin (DXR) was provided by Farmitalia-Carlo Erba, whereas Cyclosporin-A was provided by Sandoz Farma, whereas SDZ PSC 833 was gift by Novartis. Rhodamine-123 (Rho-123) and 3-(4,5-dimethylthiazol-2-yl)-2,5 diphenyl tetrazolium bromide (MTT) were purchased from Sigma (St. Luis, MO, USA). All of the working solutions were initially dissolved in dimethyl sulfoxide (DMSO) and posterior dilutions were put in culture medium.

### Cell lines and culture conditions

The lung cancer cell line INER-51 and its clone PSC-1 (non-expressing P-gp or P-gp^neg^) were grown as monolayer cultures in RPMI-1640 medium at 37°C in 5% CO_2_. INER-51 is a NSCLC cell line established in our laboratory from pleural effusion of patient diagnosed with primary lung cancer without previous chemotherapy treatment. The kidney cell line A498 (expressing P-gp) and the lung adenocarcinoma cell line INER-37 (expressing MRP1) were used as controls of P-gp function and MRP1 expression, respectively. The culture medium was supplemented with 10% FCS (Sigma, Co. St. Luis, MO, USA), 1% non-essential amino acids, 1 mM sodium pyruvate, 2 mM L-glutamine, 100 units/ml of penicillin and 100 μg/ml of streptomycin.

### Culture of monolayers and multicellular spherules

Monolayer cells were passed a week by trypsin-EDTA solution (Invitrogen™, USA). To obtain multicellular spheroids, 3.5 × 10^5 ^exponentially tumour cells were seeded in 1% agarose-coated (24-well/plate) in RPMI-1640 complete medium [14]. Cultures were routinely grown for 72 hours to acquire multicellular spheroids of approximately of 500 μm of diameter.

### P-gp non-expressing mutant clones

In order to obtain one mutant clone with the capacity to not express P-gp, INER-51 cells were treated as described by Beketic-Oreskovic, et al[15]. Briefly, 1 × 10^6 ^tumour cells were seeded in a T_25 _plastic tissue culture flask (Falcon, USA). When the cell culture achieved a semi-confluent grade, the culture medium was removed and fresh complete medium was added containing 5 μM DXR and 5 μM of SDZ PSC 833. The cells were maintained under this condition during two weeks until some survival clones were evident upon microscopic observation. A total of 6 survival clones were isolated, propagated for a month in absence of drugs, and tested for MDR1 expression by *in vitro *RT-PCR. One mutant clone that manifested stable for mdr1^neg ^phenotype was selected and cloned again. Each clone obtained was tested for MDR-1 expression again. Finally, the clone PSC-1 was further propagated and used for studies in the present communication.

### Drug resistance in monolayers or multicellular spheroids

The level of resistance to drugs was determined with the use of MTT assay as previously described by S. Cole [16] and on multicellular systems as evaluated by Furukawa, et al[17]. For monolayers, 7 × 10^3 ^cells/well were plated in 96-well/plate (Costar, USA) and drugs were added in different concentrations per well. In the case of multicellular spheroids, they were obtained as described above and were fed with fresh complete medium containing different drug concentration. After 72 hours, the culture medium was retired and MTT reagent diluted in PBS was added to obtain a final concentration of 2 mg/ml. After incubation for 4 hours, individual spheroids surrounded with formazan crystals were transferred into 1.5 ml eppendorf tubes. Cells in monolayers were washed carefully with PBS once. Both monolayers and multicellular spheroids crystals were dissolved by addition of 100% DMSO for 20 min with occasional shaking. Absorbance at 540 nm was measured using an automated microplate reader (Labsystem Multiskan MS, Finland). In each experiment, the drug determination was analysed in six individual wells. Cell survival was estimated as a percentage of the corresponding control. Drug-cytotoxicity was assayed by the IC_50_, corresponding to the 50% decrease in cell survival rate respective to not-drug treated cultures.

### Doxorubicin and Rhodamine-123 retention assays

Exponentially growing cells (3.5 × 10^5^) were seeded 72 hours prior to treatment with drugs in 1% agar-coated 24-well tissue culture plates as described above. Either multicellular spheroids or monolayer cells were incubated with increased concentrations of DXR or Rho-123 for 30 min and then washed with fresh drug-free medium twice and PBS once. Cell suspensions from monolayers were obtained by tripsinization whereas multicellular spheroids were recovered using micropipette tips. To exclude artefacts arising from DXR quenching by binding to DNA and accumulating in acidic organelles, DXR was extracted from spheroids with the method described by Wartenberg et al[18]. Finally, intracellular DXR or Rho-123 were determined by spectrofluormetry (Phototechnology, International, Princeton, New Jersey, USA) at λ_em _580 nm and λ_ex _427 or λ_em _510 nm and λ_ex _480 respectively.

### Doxorubicin efflux assays

The efflux assays were based on those previously described [19]. Briefly, monolayers or multicellular spheroids were drug loaded with DXR (30 μM) throughout incubation at 37°C in 5% CO_2 _for 30 min. The loading cells were then washed with a complete drug-free ice-cold medium and either placed on ice or incubated at 37°C in 5% CO_2 _for different intervals of time. This incubation allowed drug efflux to occur, and during experimental time the remaining drug was quantitated by spectrofluormetry as described above.

### Circumvention of P-gp-mediated efflux with Cs-A or ATP-depletion

For evaluation of P-gp activity, monolayers or multicellular spheroids were pre-incubated for 1 hour with increasing drug concentrations of cyclosporin-A (Cs-A). After pre-incubation, multicellular spheroids were loaded for 30 min with 30 μM of DXR in presence of the reversal agent and afterwards the DXR uptake was assessed again. For ATP-depletion, cells were pre-incubated for 20 min at 4°C in PBS/BSA (1 mg/ml) containing 1 mM sodium-cyanide, 10 mM sodium-fluoride and 10 mM sodium azide prior to the addition of DXR. After centrifugation at 1000 × g for 15 min, both monolayers and multicellular spheroids were loaded with different DXR concentrations in the presence of ATP synthesis inhibitors and the fluorescence was measured as mentioned above.

### *In vitro *Reverse transcriptase-PCR assay

Total RNA was extracted from the cell lines with Trizol reagent (Invitrogen™, USA) according to manufacturer's instructions. Single stranded cDNA was synthesized by reverse transcription from 5 μg of total RNA using Superscript™ RNAse Reverse Transcriptase (Invitrogen™, USA) and oligo-dT_16–18_. The amplification was performed in a final volume of 25 μl, containing 0.5 μl cDNA, 50 pM of each oligonucleotide primer, 30 μM of each dNTPs, 2.5 units of Taq DNA polymerase, 1.5 mM MgCl_2_, 20 mM Tris-HCl (pH 8.4) and 50 mM KCl. Amplification was carried out in a Thermal Cycler (Programmed Thermal Controller, model PTC-100, MJ Research Inc., USA) for 35 cycles of denaturalisation at 94°C for 1 min, annealing at 55–60°C for 2 min, and polymerisation at 72°C for 3 min. The PCR primers and expected product size were as follows: For MDR-1, forward: 5'-cccatcattgcaatagcagg-3' and reverse: 5'-gttcaaacttctgctcctga-3 [150 bp]; MRP1, forward: 5'-tctctcccgacatgaccgagg-3' and reverse: 5'-ccaggaatatgatgccccgacttc-3' [140 bp]; topoisomerase IIα, forward: 5'-tttaaggcccaagtccagttaaac-3' and reverse: 5'-gtataacaatatcatcaagattgt [343 pb]; topoisomerase IIβ, forward: 5'-gaagtgttcactagtaaaatacagt-3' and reverse: 5'-cataatctttccatagcgtaaggtt-3' [336 bp]; topoisomerase I, forward: 5'-aagcagaggaagtagctacg-3' and reverse: 5'-gctcatctgtttccgagctt-3' [206 bp]; GST-μ, forward: 5'-gaactccctgaaaagctaaag-3' and reverse: 5'-gttgggctcaaatatacggtgg-3' [250 bp]; G3PDH, forward: 5'-tggggaaggtgaaggtcgga-3' and reverse: 5'-gaaggggtcattgatggcaa-3' [110 bp].

## List of abbreviations

Cs-A: Cyclosporin-A

DXR: Doxorubicin

MCR: Multicellular resistance

MTT: 3-(4,5-dimethylthiazol-2-yl)-2,5 diphenyl tetrazolium bromide

NSCLC: Non-small cell lung cancer

P-gp: P-Glycoprotein

Rho-123: Rhodamine 123

MDR: Multidrug Resistance

DMSO: Dimethyl sulfoxide

## Authors' contributions

All author(s) contributed equally to this work.
